# Characteristics of Cortical Atrophy and White Matter Lesions Between Dementia With Lewy Bodies and Alzheimer's Disease: A Case-Control Study

**DOI:** 10.3389/fneur.2021.779344

**Published:** 2022-01-11

**Authors:** Han Zhu, Hao Lu, Fei Wang, Shuai Liu, Zhihong Shi, Jinghuan Gan, Xiaoshan Du, Yaqi Yang, Daibin Li, Lichen Wang, Yong Ji

**Affiliations:** ^1^Clinical College of Neurology, Neurosurgery and Neurorehabilitation, Tianjin Medical University, Tianjin, China; ^2^Department of Radiology, Tianjin Huanhu Hospital, Tianjin, China; ^3^Department of Neurology, Tianjin Huanhu Hospital, Tianjin, China; ^4^Tianjin Key Laboratory of Cerebrovascular and of Neurodegenerative Diseases, Tianjin Dementia Institute, Tianjin, China; ^5^Department of Neurology, Beijing Tiantan Hospital, Capital Medical University, Beijing, China; ^6^China National Clinical Research Center for Neurological Diseases, Beijing, China

**Keywords:** dementia with Lewy bodies, Alzheimer's disease, magnetic resonance imaging, medial temporal atrophy, posterior atrophy, Fazekas scale

## Abstract

**Introduction:** Currently, there is still clinical overlap between dementia with Lewy bodies (DLB) and Alzheimer's disease (AD) patients, which may affect the accuracy of the early diagnosis of DLB. For better diagnosis and prognosis, further exploration of local cortical atrophy patterns and white matter lesions is needed.

**Methods:** We reviewed the outpatient medical records of 97 DLB patients and 173 AD patients from January 2018 to September 2020 along with 30 matched outpatient clinic normal elderly people. MRI visual rating scales, including medial temporal lobe atrophy (MTA), global cortical atrophy-frontal subscale (GCA-F), posterior atrophy (PA), Fazekas scale, Evans Index and cerebral microbleeds were evaluated and analyzed in DLB and AD patients with different severities and normal controls.

**Results:** Overall, patients with DLB had higher scores on all visual rating scales than the normal controls. Meanwhile, compared with AD, DLB had lower MTA scores in the mild to moderate groups (both *p* ≤ 0.001), but the GCA-F and PA scores were similar (all *p* > 0.05). The Fazekas scores in the moderate to severe DLB group were lower than those in the AD group (*p* = 0.024 and *p* = 0.027, respectively). In addition, the diagnostic performance and sensitivity of multiple imaging indicators for DLB were better than that of MTA alone (the combination of MTA, GCA-F, PA, Fazekas visual rating scales, AUC = 0.756, 95%CI: 0.700–0.813, sensitivity = 0.647, specificity = 0.804 and MTA visual rating scale, AUC = 0.726, 95%CI: 0.667–0.785, sensitivity = 0.497, specificity = 0.876, respectively).

**Conclusion:** The medial temporal lobe of DLB patients was relatively preserved, the frontal and parietal lobes were similarly atrophied to AD patients, and the white matter hyperintensity was lighter than that in AD patients. Combined multiple visual rating scales may provide a novel idea for the diagnosis of early DLB.

## Introduction

Dementia is a progressive deterioration in cognitive ability and progressive decline in the capacity of daily independent living (1). At present, the incidence of dementia with Lewy bodies (DLB) ranks second among dementia, second only to Alzheimer's disease (AD), accounting for ~10–20% of dementia (2, 3). The histopathological features of DLB are characterized by the accumulation and aggregation of α-synuclein, such as Lewy bodies and Lewy neurites, in the brainstem, subcortical, limbic, and neocortical regions. However, the underlying pathology is sometimes mixed with AD-related pathologies such as extracellular β-amyloid plaques, senile plaques with tau-positive neurites, and neurofibrillary tangles that commonly occur in DLB (4). The core clinical features of DLB include fluctuating cognitive function, recurrent visual hallucinations, parkinsonism, and RBD (rapid eye movement [REM] sleep behavior disorder) (4, 5). AD is the single largest cause of dementia and is characterized by gradual cognitive decline and progressive cerebral atrophy (6). The gradual intraneuronal accumulation of neurofibrillary tangles formed as a result of abnormal hyperphosphorylation of cytoskeletal tau protein, extracellular deposition of amyloid β (Aβ) protein as senile plaques, and massive neuronal death are important neuropathological hallmarks of AD (7). Many studies have shown that the core symptoms of DLB, such as RBD and visual hallucinations, are also present in AD patients (8, 9). Because of the overlap between these clinical symptoms, the diagnostic criteria for DLB are high specificity and low sensitivity (10). With the development of pathological studies, it is now recognized that co-pathology can be present in neurodegenerative diseases (11). Several autopsy studies have shown the co-existing of AD pathology may be in DLB patients, and conversely Lewy bodies (LBs) pathology can also be observed in some AD patients (12, 13). There is a strong association between cortical α-syn pathology and Aβ plaque burden in DLB patients with AD co-pathology (14). For patients with confirmed AD when accompanied by LBs pathology, there could be performances related to core symptoms of DLB as well as neuropsychiatric symptoms (15, 16). Therefore, the co-pathology in the brains of AD and DLB patients may also be a possible reason for overlapping symptoms seen in patients.

Visual rating scales are simple and quick and can evaluate general and focal cerebral atrophy in patients with cognitive impairment. Some literature has confirmed that the sensitivity and specificity of medial temporal lobe atrophy (MTA) visual rating scale range in the diagnosis of AD, other types of dementia, and NC were 62–90 and 67–92%, respectively (17–20). Later, other studies also compared the sensitivity and specificity of the posterior atrophy (PA) visual rating scale in distinguishing between AD, DLB, and NC ranging from 58–85 to 46–72%, respectively (20, 21). In view of the effectiveness of visual rating scales, they are widely used in the clinic to improve the accuracy of clinical diagnosis. Currently, medial temporal lobe atrophy is the diagnostic criterion for AD (22), preserved medial temporal lobe volume is a supportive biomarker of DLB (5). However, some studies have shown that medial temporal lobe atrophy also occurs in DLB. It may affect the practicability of the MTA for individual judgments (22, 23). Therefore, it is necessary to combine the MTA scale with other visual rating scales to improve the accuracy of diagnosis (19). Many previous studies have demonstrated that white matter hyperintensities (WMHs) are more common in patients with AD than in normal people (24, 25), but there is still controversy regarding WMHs in patients with DLB. Some studies have suggested that WMHs are more severe in DLB patients than in AD patients or healthy controls (24–26). On the contrary, other studies have not found significant differences between them (27, 28). With the development of automated analysis methods, it was found that the visual rating scale and automatic analysis had high accuracy and over 75% sensitivity and specificity in assessing medial temporal lobe volumes. These results are highly consistent (29, 30).

Previous studies were limited to the comparison of individual brain structures or involved studies of white matter lesions that only focused on AD. Therefore, we conducted this study and aimed to explore brain atrophy patterns and white matter lesions in different regions of DLB patients and AD patients to improve the differential diagnosis of the two diseases.

## Materials and Methods

### Participants

This study was a case-control study, a total of 1,412 subjects were seen by cognitive impairment clinical services in Huanhu Hospital, Tianjin, China, from January 2018 to September 2020.

The DLB diagnosis was based the current criteria (5) and required dementia plus 2 of 4 clinical features (visual hallucinations, fluctuations, parkinsonism, and REM sleep behavior disorder) (5). The diagnosis of AD was made on the criteria of “probable AD” in the National Institute of Neurological Disorders and Stroke-Alzheimer Disease and Related Disorders (NINCDS-ADRDA) AD diagnostic criteria (31), In addition, due to the lack of pathological studies confirmed, we excluded patients with possible concomitant core clinical features of DLB when enrolling patients with AD, to avoid the possibility of patients with AD tend to convert to DLB with the progression in clinical manifestations. The inclusion criteria for all patients were as follows: (1) age 45 years and above; (2) at least one caregiver or relative accompanying the patient; and (3) voluntary participation in the study. The exclusion criteria were as follows: (1) consciousness disorder, severe aphasia or hemiplegia; the neuropsychological test could not be completed; (2) comorbidity of malignant tumor, connective tissue disease, blood disease, malnutrition, etc; (3) previous history of mental illness; and (4) refusal to participate.

**Figure 1:**
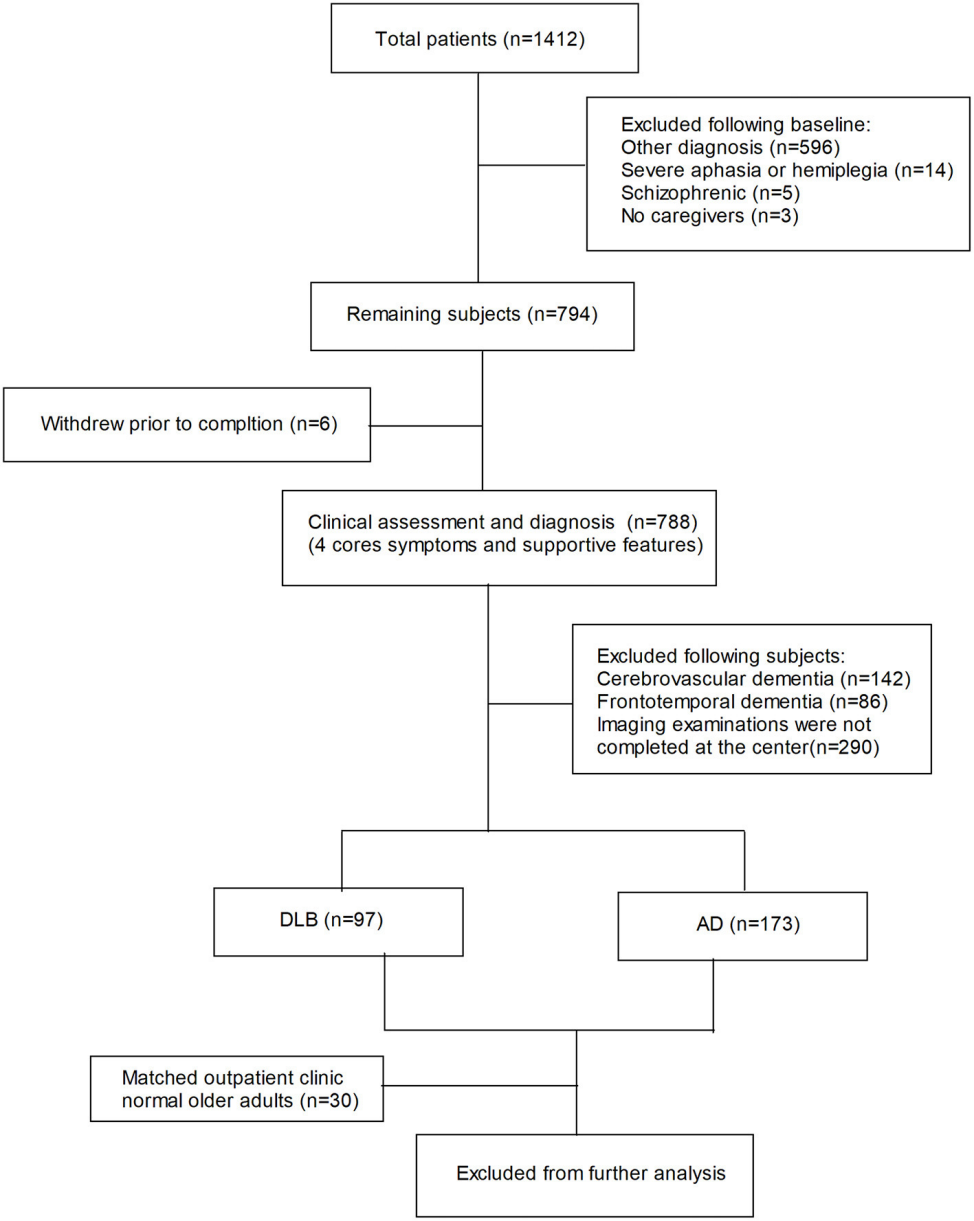
Flow diagram. Alzheimer's disease; DLB, dementia with Lewy bodies.

Of the 1,412 total patients, 1,142 were ultimately excluded. Of the initial exclusions, 596 were due to other diagnoses, 14 due to severe aphasia or hemiplegia, 5 due to schizophrenia, and 3 due to lack of caregiver. Of the 794 patients remaining after the initial exclusion, 6 withdrew prior to completion. The remaining 788 subjects had their assessment and diagnosis refined. In addition, 142 were ultimately excluded because of cerebrovascular dementia, 86 due to frontotemporal dementia, and 290 due to failure to complete imaging or rating at our center or missed images. Finally, 97 were classified as DLB, 173 were classified as AD, and 30 outpatient clinic normal older adults were matched as healthy controls, and they did not differ significantly in age, gender, or education from those with AD and DLB (Figure 1).

This study was designed and conducted in accordance with the Declaration of Helsinki, and written informed consent was obtained from all participants.

### Neuropsychological Assessment

All participants underwent a routine clinical assessment, including the collection of basic information, detailed history, mental state examination, neurological examination, laboratory tests and neuroimaging. Among them, a detailed medical history includes hypertension, type 2 diabetes mellitus (T2DM), cardiovascular disease (including the history of cardiac arrest, atrial fibrillation, coronary artery disease and congestive heart failure), hyperlipidemia, orthostatic hypotension (in this study we defined OH as a reduction in systolic BP of at least 30 mmHg and/or diastolic BP of at least 15 mmHg within 30 to 180 seconds of active standing without significant heart rate changes in subjects), and syncope (at least 1 attack). The neuropsychological assessment mainly included the Chinese Mini-mental State Examination (C-MMSE), Montreal Cognitive Assessment Test (MoCA), and Clinical Dementia Rating (CDR) score. The CDR was used to determine the severity of the disease with the following scores: 0.5–1.0, mild; 2, moderate; and 3, severe (32). The scale evaluation was completed by dementia scale evaluators trained and certified by the neuropsychological scale.

### MRI Study

#### MRI Parameters

All participants underwent diagnostic multisequence MRI, which included a sagittal 3D T1-weighted gradient-echo sequence (TR = 11 ms, TE = 4.94 ms, Flip angle = 15°, Image matrix = 232^*^256, Averages = 3, Concatenations = 1), a transverse T2-weighted fluid-attenuated inversion-recovery (FLAIR) sequence (TR = 8570 ms, TE = 95 ms, Flip angle = 130°, Image matrix = 218^*^256, Averages = 1, Concatenations = 2) and a susceptibility-weighted imaging (SWI) sequence (TR = 28 ms, TE = 20 ms, Flip angle = 15°, Image matrix = 221^*^320, Averages = 1, Concatenations = 1). All MRI was performed with whole-brain coverage. The data was acquired from a SIEMENS TRIO scanner (3.0 Tesla).

Multiplanar oblique coronal (perpendicular to the axis of the hippocampus), transverse and coronal position reconstructions were made of 3D T1-weighted images.

#### MRI Readings

**Figure 2:**
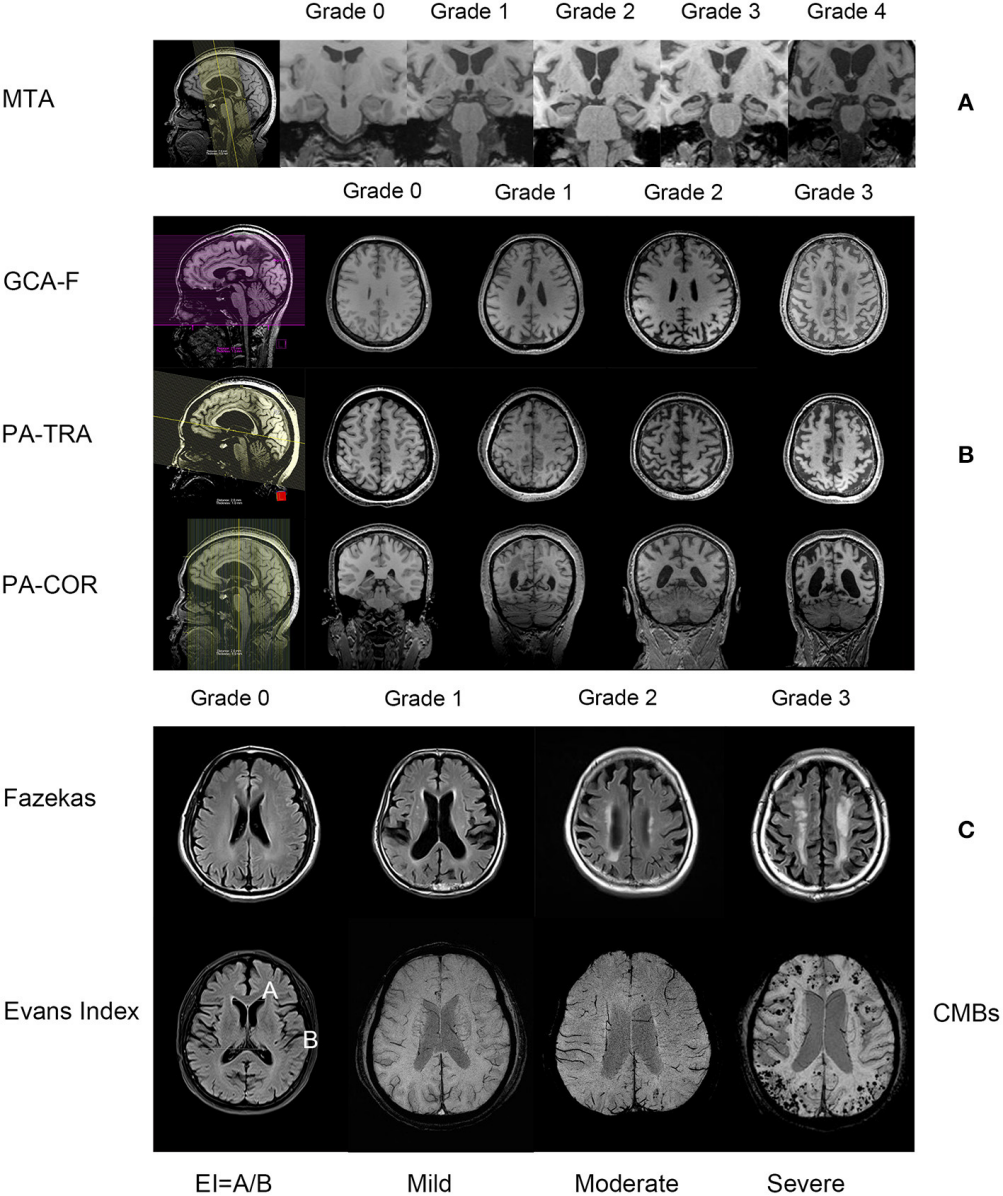
The reconstruction mode and the description of the degree of the MRI visual rating scales (all images are from subjects in this study). **(A)** contains the MTA visual rating scale, from left to right are the positioning phase, grade 0–4 (3D T1-weighted images, oblique coronal); **(B)** contains the GCA-F, PA visual rating scale, from left to right are the positioning phase, grade 0–3 (GCA-F is the transverse section of T1-weighted images, PA is the transverse section and coronal section of T1-weighted images); **(C)** contains the Fazekas visual rating scale, from left to right are grade 0–3 (T2-weighted fluid-attenuated inversion-recovery sequence), the Evans Index, the value is the ratio between the maximal diameter of the anterior horns of the lateral ventricles-A and the greatest internal diameter of the skull-B, and CMBs, according to the number of microbleeds, from left to right are mild, moderate and severe (the figure shows the susceptibility-weighted imaging sequence, the 3D T1-weighted gradient-echo sequence is not shown). MTA, medial temporal lobe atrophy; GCA-F, global cortical atrophy-frontal subscale; PA, posterior atrophy; CMBS, cerebral microbleeds.

All of the MRI readings were performed by three experienced neuroradiologists. If two or more neuroradiologists provided the same scores for a given MRI, they were the final rating results. If the scores of the three were different from each other, the subject was excluded. The three neuroradiologists were blinded to the diagnosis and demographic and clinical information. The visual rating scales included medial temporal lobe atrophy (MTA), global cortical atrophy-frontal subscale (GCA-F), posterior atrophy (PA), Fazekas scale, Evans Index (EI) and cerebral microbleeds (CMBs) (Figure 2). Among them, the MTA was scored separately for the left and right sides, and the overall MTA score was obtained by calculating the average of both sides.

MTA: The MTA scale scores the degree of atrophy from 0 to 4 in the hippocampus, parahippocampal gyrus, entorhinal cortex and the surrounding cerebrospinal fluid spaces. The scores were as follows: grade 0, no atrophy; grade 1, only widening of choroid fissure; grade 2, additional widening of the temporal horn of lateral ventricle; grade 3, moderate loss of hippocampal volume (decrease in height); and grade 4, severe volume loss of hippocampal volume (18).

GCA-F: The GCA-F scale scores the degree of atrophy from 0 to 3 in frontal cortex atrophy and sulcal dilatation. The scores were as follows: grade 0, no cortical atrophy; grade 1, mild atrophy (opening of sulci); grade 2, moderate atrophy (volume loss of gyri); and grade 3, severe atrophy “knife blade atrophy” (33).

PA: The PA scale scores the degree of atrophy from 0 to 3 in the posterior cingulate sulcus, precuneus, parieto-occipital sulcus and parietal cortex. The scores were as follows: grade 0, no cortical atrophy (closed posterior cingulate and parieto-occipital sulcus and closed sulci of the parietal lobes and precuneus); grade 1, mild widening of the posterior cingulate- and parieto-occipital sulcus, with mild atrophy of the parietal lobes and precuneus; grade 2, substantial widening of the posterior cingulate and parieto-occipital sulcus, with substantial atrophy of the parietal lobes and precuneus; and grade 3, end-stage atrophy with the evident widening of both sulci and knife-blade atrophy of the parietal lobes and precuneus (34).

Fazekas scale: The Fazekas scale scores a degree from 0 to 3 to reflect the whole white matter lesions. The scores were as follows: grade 0, no or single punctate lesion; grade 1, multiple punctate lesions; grade 2, beginning confluency of lesions (bridging); and grade 3, large confluent lesions (35).

EI: The Evans Index is the ratio between the maximal diameter of the anterior horns of the lateral ventricles (A) and the greatest internal diameter of the skull (B). Values > 0.3 are considered pathological (36). We divided these values into 3 scores as follows: 0 score, values < 0.3; 1 score, 0.3 < values < 0.35; 2 scores, values > 0.35.

CMBs: In the T2 gradient echo sequence or SWI, there were round or quasi round areas with clear boundaries and black or low signal areas with a diameter of 2–10 mm. Regarding the grading of the CMB, we classified the degree of severity according to the number of microbleeds as absent, mild (1 to 5), moderate (6 to 15) or severe (>15) (37, 38).

### Statistical Analysis

Quantitative variables (age, scores on the C-MMSE, MoCA, CDR, and visual rating scales) were presented as mean ± standard deviation (SD) when the data was normally distributed and the median (Q_25,75_) for non-normally distributed data. Categorical data (gender, education, smoking, hypertension, T2DM, cardiovascular disease, hyperlipidemia, OH and syncope) were presented as frequency counts and percentages. The Chi-square tests were used for categorical variable comparisons, and independent-sample *T*-tests or ANOVA were used for the normally distributed continuous variable comparisons, and the non-parametric Mann-Whitney *U-*test and Kruskal-Wallis tests were used for non-normally distributed statistical variable comparisons. To evaluate the diagnostic performance of visual rating scales, the receiver operating characteristic (ROC) curves were drawn with 1-specificity and sensitivity as abscissa and ordinate respectively.

All data were descriptively analyzed using IBM SPSS Statistics software (version 22). A *p*-value < 0.05 was considered statistically significant in this study.

## Results

### General Characteristics and Neuropsychological Assessment of the Study Subjects

A total of 300 research subjects were included, 173 patients with probable AD (70 males, 103 females), 97 with probable DLB (44 males, 53 females) and 30 normal controls (16 males, 14 females). The general clinical characteristics and neuropsychological assessment of the subjects were compared in Table 1. The average age at first evaluation in the AD group was 69.60 ± 9.46 years, the average age at first evaluation in the DLB group was 72.22 ± 7.30 years, and that in the control group was 69.8 ± 6.75 years. There was no significant difference in age, gender, education level, smoking, hypertension, T2DM, cardiovascular disease, hyperlipidemia, OH or syncope prevalence between AD and DLB patients and NCs (all *p* > 0.05). The C-MMSE and MOCA scores were analyzed between AD and DLB patients, and there was no significant difference between the two groups (both *p* > 0.05).

**Table 1:** General clinical characteristics of each subject group.

**Subjects characteristic**	**AD**	**DLB**	**NC**	***p* value**
	**Mean ± SD or median (Q_**25,75**_)**	**Mean ± SD or median (Q_**25,75**_)**	**Mean ± SD or median (Q_**25,75**_)**	
**Number (%)**	173 (57.7%)	97 (32.3%)	30 (10%)	
**Gender**				0.374
Male Female	70 103	44 53	16 14	
**Age**	69.60 ± 9.66	72.22 ± 7.30	69.8 ± 6.75	0.256
**Education (years)**				0.51
≤ 6	53	24	8	
7-12	70	48	16	
≥12 **Smoking (%)** **Hypertension (%)**	49 34 (19.7%) 54 (31.2%)	24 25 (25.8%) 39 (40.2%)	6 6 (20.0%) 6 (20.0%)	0.49 0.09
**T2DM (%)**	12 (6.9%)	14 (14.4%)	3 (10.0%)	0.135
**Cardiovascular disease (%)** Cardiac arrest Atrial fibrillation Coronary artery disease Congestive heart failure **Hyperlipidemia (%)** **OH (%)** **Syncope**	2 (1.2%) 4 (2.3%) 25 (14.5%) 6 (3.5%) 20 (11.6%) 8 (4.6%) 2 (1.2%)	1 (1.0%) 1 (1.0%) 18 (18.6%) 4 (14.1%) 11 (11.3%) 8 (8.2%) 2 (2.1%)	0 (0%) 0 (0%) 3 (10.0%) 1 (3.3%) 2 (6.7%) 1 (3.3%) 0 (0%)	0.841 0.522 0.464 0.958 0.725 0.393 0.658
**C-MMSE**	15.0 (8.0, 19.0)	16.0 (11.0, 21.0)	29.0 (28.0, 30.0)	0.446
**MOCA**	9.0 (4.0, 14.75)	9.0 (6.0, 15.0)	28.0 (27.0, 29.0)	1

*AD, Alzheimer's disease; DLB, dementia with Lewy bodies; NC, normal controls; T2DM, type 2 diabetes mellitus; OH, orthostatic hypotension; C-MMSE, Chinese mini-mental state examination; MoCA, Montreal cognitive assessment; Quantitative variables (age, scores on the C-MMSE and MoCA) are presented as mean ± standard deviation (SD) when the data were normally distributed and the median (Q_25,75_) for non-normally distributed data. Categorical data (gender, education, smoking, hypertension, T2DM, cardiovascular disease, hyperlipidemia, OH and syncope) are presented as frequency counts and percentages. The C-MMSE and MoCA data compared statistical results between AD and DLB subjects; Other data compared statistical results between AD, DLB, and NC subjects*.

### MRI Visual Rating Scales in AD, DLB, and NC Groups

The MRI visual rating scales of each group are shown in Table 2. There was no significant difference between the left and right scores of the MTA group (AD and DLB, left vs. right, *p* = 0.811 and *p* = 0.688, respectively), indicating that there was no significant difference in the atrophy degree between the left and right medial temporal lobes. In addition to the Evans Index, all the visual rating scales were significantly different (*p* = 0.055 and others *p* < 0.001, respectively). The further between-group analysis found that the AD group had significantly higher scores on all the visual rating scales than the control group (AD vs. NC, all *p* < 0.001). At the same time, compared with the control group, the DLB group also had significantly higher scores on all the visual rating scales (DLB vs. NC, CMBs *p* = 0.018 and others *p* < 0.001, respectively). However, compared with AD, DLB had lower MTA, PA and Fazekas scores (AD vs. DLB, *p* < 0.001, *p* = 0.003 and *p* = 0.002, respectively), but the groups did not differ significantly in GCA-F and CMB scores (AD vs. DLB, *p* = 0.711 and *p* = 0.065, respectively).

**Table 2:** Neuropsychological assessment scores and MRI visual rating scales of each subject group.

**Visual rating scales**	**AD**	**DLB**	**NC**	**Total** ***p* value**	**AD vs. NC** ***p* value**	**DLB vs. NC *p* value**	**AD vs. DLB *p* value**
	**Median (Q_**25,75**_)**	**Median (Q_**25,75**_)**	**Median (Q_**25,75**_)**				
MTA	2.0 (1.5, 3.0)	1.0 (1.0, 2.0)	0 (0, 1.0)	< 0.001^***^	< 0.001^***^	< 0.001^***^	< 0.001^***^
Left	2.0 (1.5, 3.0)	1.0 (1.0, 2.0)					
Right	2.0 (1.5, 3.0)	1.0 (1.0, 2.0)					
GCA-F	1.0 (1.0, 2.0)	1.0 (1.0, 2.0)	0 (0, 0.25)	< 0.001^***^	< 0.001^***^	< 0.001^***^	0.711
PA	2.0 (1.0, 2.0)	1.0 (1.0, 2.0)	0 (0)	< 0.001^***^	< 0.001^***^	< 0.001^***^	0.003^**^
Fazekas	2.0 (1.0, 3.0)	1.0 (1.0, 2.0)	0 (0)	< 0.001^***^	< 0.001^***^	< 0.001^***^	0.002^**^
Evans index	0 (0)	0 (0)	0 (0)	0.055			
CMBs	0 (0, 1.0)	0 (0, 1.0)	0 (0)	< 0.001^***^	< 0.001^***^	0.018^*^	0.065

*AD, Alzheimer's disease; DLB, dementia with Lewy bodies; NC, normal controls; MTA, medial temporal lobe atrophy; GCA-F, global cortical atrophy-frontal subscale; PA, posterior atrophy; CMBs, cerebral microbleeds. The data results for non-normally distributed quantitative variables (scores on visual rating scales) are described by the median (Q_25,75_)*.

* *p < 0.05*,

** *p < 0.01*,

*** *p < 0.001*.

### MRI Visual Rating Scales in the AD and DLB Groups Under the Different Severities

**Figure 3:**
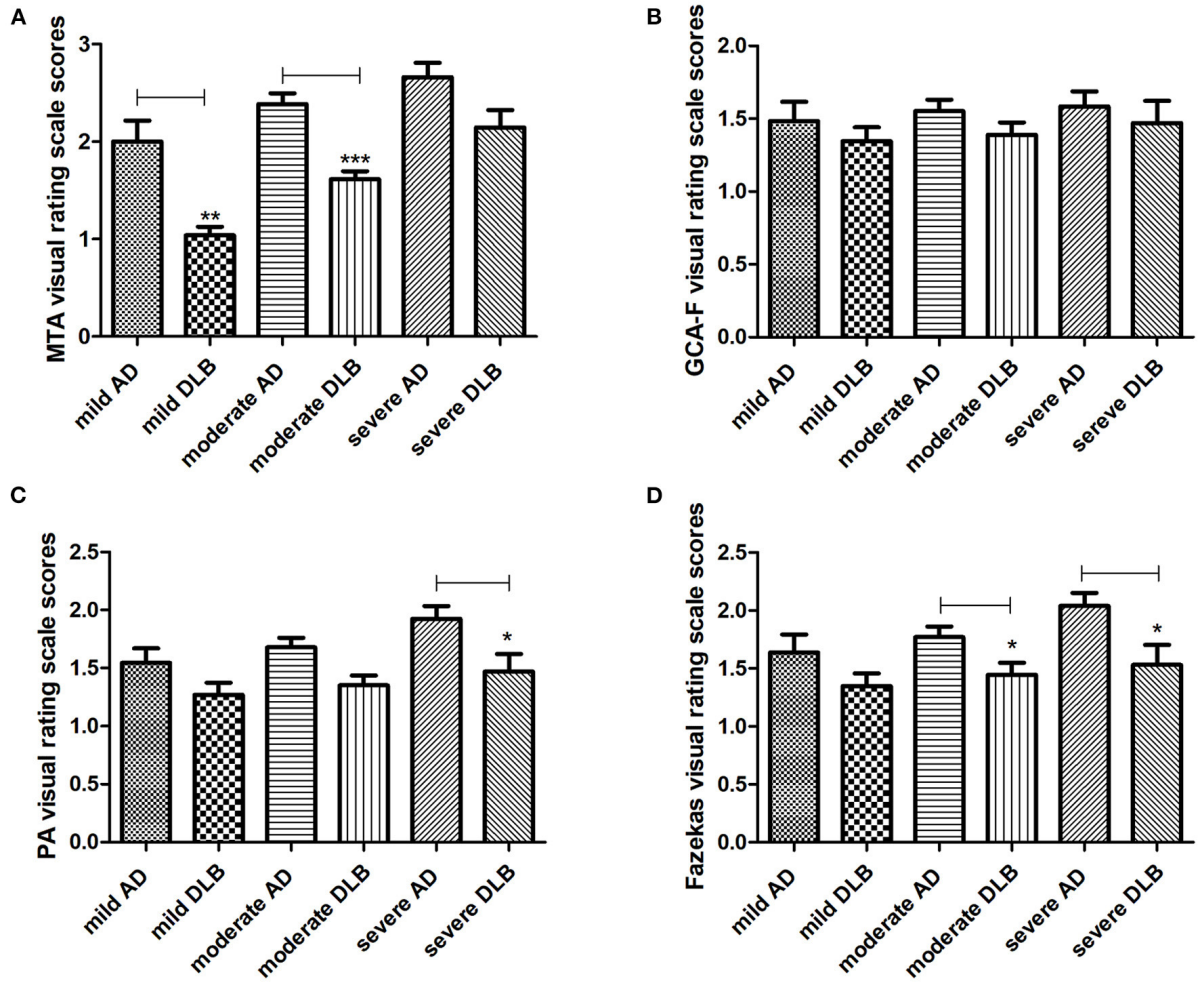
MRI visual rating scales in AD and DLB subjects. The results of visual rating scale scores between AD and DLB groups under the same severity are described in **(A–D)**. AD, Alzheimer's disease; DLB, dementia with Lewy bodies; MTA, medial temporal lobe atrophy; GCA-F, global cortical atrophy-frontal subscale; PA, posterior atrophy. *The results showed significant differences between AD and DLB. ^*^p < 0.05, ^**^p < 0.01, ^***^p < 0.001.

The MRI visual rating scales of the AD and DLB groups at different severities are shown in Table 3 and Figure 3. Compared with AD, DLB had lower MTA scores in the mild to moderate groups (both *p* ≤ 0.001), but the GCA-F and PA scores were similar (all *p* > 0.05). In comparison between the severe AD and DLB groups, the PA score was lower in the DLB group than in the AD group (*p* = 0.037), but the MTA and GCA-F atrophy scores were similar (both *p* > 0.05). A further novel finding was that the Fazekas scores in the moderate to severe DLB group were lower than those in the AD group (*p* = 0.024 and *p* = 0.027, respectively). However, there were no significant differences in the Evans Index and CMB visual rating scales between the AD and DLB groups (all *p* > 0.05).

**Table 3:** MRI visual rating scales in AD and DLB subjects with different severities.

**Visual rating scales**	**AD**			**DLB**			**Mild** ***p* value**	**Moderate** ***p* value**	**Severe** ***p* value**
	**Median (Q**_**25,75**_ **)**			**Median (Q**_**25,75**_ **)**					
	**Mild**	**Moderate**	**Severe**	**Mild**	**Moderate**	**Severe**			
Number	33	87	53	26	54	17			
MTA	2.0 (1.0, 3.0)	2.0 (1.5, 3.0)	2.5 (2.0, 4.0)	1.0 (1.0, 1.0)	1.75 (1.0, 2.0)	2.0 (1.75, 3.0)	0.001^**^	<0.001^***^	0.072
GCA-F	1.0 (1.0, 2.0)	1.0 (1.0, 2.0)	2.0 (1.0, 2.0)	1.0 (1.0, 2.0)	1.0 (1.0, 2.0)	2.0 (1.0, 2.0)	0.321	0.182	0.57
PA	2.0 (1.0, 2.0)	1.0 (1.0, 2.0)	2.0 (1.0, 2.0)	1.0 (1.0, 2.0)	1.0 (1.0, 2.0)	2.0 (1.0, 2.0)	0.105	0.092	0.037^*^
Fazekas	1.0 (1.0, 2.0)	2.0 (1.0, 2.0)	2.0 (1.0, 3.0)	1.0 (1.0, 2.0)	1.0 (1.0, 2.0)	2.0 (1.0, 2.0)	0.184	0.024^*^	0.027^*^
Evans index	0 (0)	0 (0, 1.0)	0 (0, 1.0)	0 (0, 0.25)	0 (0, 1.0)	0 (0, 0.5)	0.584	0.844	0.67
CMBs	0 (0, 1.0)	0 (0)	0 (0)	0 (0)	0 (0)	0 (0)	0.453	0.759	0.613

*AD, Alzheimer's disease; DLB, dementia with Lewy bodies; MTA, medial temporal lobe atrophy; GCA-F, global cortical atrophy-frontal subscale; PA, posterior atrophy; CMBs, cerebral microbleeds. The table shows the statistical results between AD and DLB subjects with the same severity. The data results for non-normally distributed quantitative variables (scores on visual rating scales) are described by the median (Q_25,75_)*.

* *p < 0.05*,

** *p < 0.01*,

*** *p < 0.001*.

### The ROC Analyses for Diagnostic Performances

**Figure 4:**
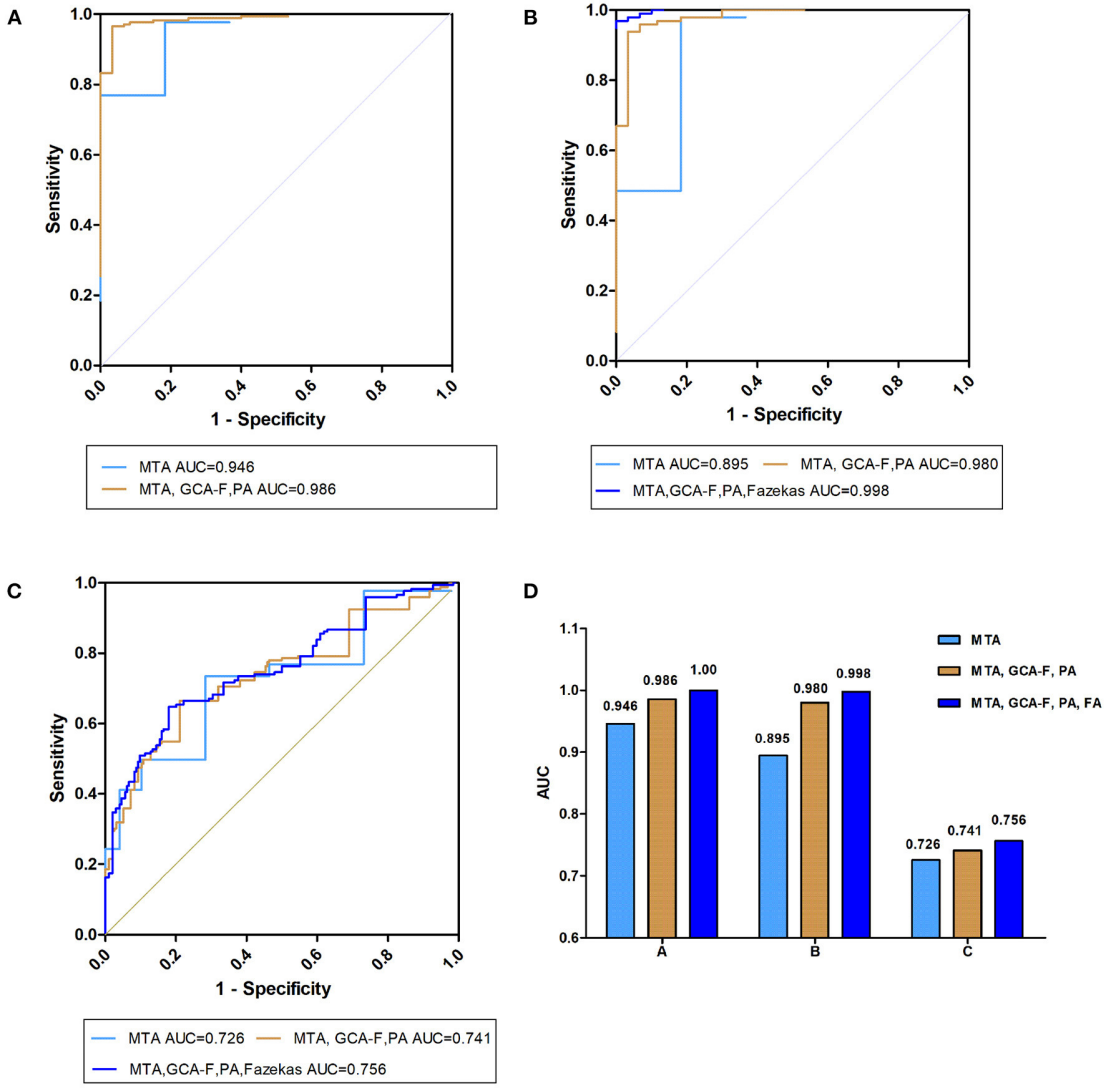
Prediction of visual rating scale diagnostic performances. Receiver operating characteristic (ROC) curves presenting the predicting capacity of “visual rating scale” in **(A)** AD vs. NC, **(B)** DLB vs. NC, and **(C)** AD vs. DLB different research groups through the MTA visual rating scale (light blue curve), MTA, GCA-F and PA visual rating scale (brown curve) and the three above combined with Fazekas visual rating scales (dark blue curve). AD, Alzheimer's disease; DLB, dementia with Lewy bodies; NC, normal controls; AUC, area under the curve; MTA, medial temporal lobe atrophy; GCA-F, global cortical atrophy-frontal subscale; PA, posterior atrophy.

To diagnose AD and DLB, MTA visual rating scale and multiple visual rating scales were analyzed. The areas under the ROC curve (AUCs) for MTA visual rating scale, the combination of three visual rating scales (MTA, GCA-F and PA visual rating scales) and the combination of four visual rating scales (MTA, GCA-F, PA and Fazekas visual rating scales) in AD and DLB are shown in Table 4 and Figure 4. Compared with NC, AD and DLB diagnoses had the highest AUCs for the combination of four visual rating scales (AD vs. NC, AUC = 1.00, sensitivity = 1.00, specificity = 1.00 and DLB vs. NC, AUC = 0.998, 95%CI: 0.994–1.00, sensitivity = 0.969, specificity = 1.00, respectively), they were superior to the combination of three (AD vs. NC, AUC = 0.986, 95%CI: 0.971–1.00, sensitivity = 0.971, specificity = 0.933 and DLB vs. NC, AUC = 0.980, 95%CI: 0.959–1.00, sensitivity = 0.959, specificity = 0.933, respectively) and MTA alone (AD vs. NC, AUC = 0.946, 95% CI: 0.915–0.978, sensitivity = 0.769, specificity = 1.00 and DLB vs. NC, AUC = 0.895, 95%CI: 0.833–0.957, sensitivity = 0.979, specificity = 0.633, respectively). Similar results were obtained for the analysis of AD and DLB, the AUCs for the combination of four and three visual rating scales were better than MTA alone (AD vs. DLB, AUC = 0.756, 95%CI: 0.700–0.813, sensitivity = 0.647, specificity = 0.804, AUC = 0.741, 95%CI: 0.683–0.799, sensitivity = 0.665, specificity = 0.742 and AUC = 0.726, 95%CI: 0.667–0.785, sensitivity = 0.497, specificity = 0.876, respectively). Meanwhile, there were significant statistical differences in the AUC values between different visual rating scales in diagnosing AD and DLB (all *p* < 0.001).

**Table 4:** The ROC analysis results of different diagnostic indicators.

**Diagnostic indicators**	**AD vs. NC**			**DLB vs. NC**			**AD vs. DLB**		
	**AUC 95%CI**	**SE**	**SP**	**AUC 95%CI**	**SE**	**SP**	**AUC 95%CI**	**SE**	**SP**
MTA	0.946^***^ (0.915–0.978)	0.769	1.000	0.895^***^ (0.833–0.957)	0.979	0.633	0.726^***^ (0.667–0.785)	0.497	0.876
MTA, GCA-F, PA	0.986^***^ (0.971–1.000)	0.971	0.933	0.980^***^ (0.959–1.000)	0.959	0.933	0.741^***^ (0.683–0.799)	0.665	0.742
MTA, GCA-F, PA, Fazekas	1.000^***^	1.000	1.000	0.998^***^ (0.994–1.000)	0.969	1.000	0.756^***^ (0.700–0.813)	0.647	0.804

*AD, Alzheimer's disease; DLB, dementia with Lewy bodies; NC, normal controls; AUC, area under the curve; 95%CI, 95% confidence interval; SE, sensitivity; SP, specificity; MTA, medial temporal lobe atrophy; GCA-F, global cortical atrophy-frontal subscale; PA, posterior atrophy*.

*** *p < 0.001*.

## Discussion

In this study, the MRI visual rating scales were used to analyze brain structural characteristics between DLB and AD. The diagnostic performances of visual rating scales and their relationship with cognitive situations were explored. The main findings were: (1) The patients with cognitive impairment have more obvious brain atrophy and white matter hyperintensity than those with normal cognition; (2) Compared with AD patients, patients with mild to moderate DLB have relatively preserved medial temporal structures, accompanied by similar frontal and parietal atrophy. At the same time, atrophy of the medial temporal lobe and frontal lobe in severe DLB patients tended to be consistent with that in severe AD patients, but parietal lobe atrophy was more severe in AD patients. AD patients have more severe white matter lesions than DLB patients; (3) For the diagnosis of DLB, the multiple imaging indicators combined AUCs were the highest indicating that a normal MTA score is important, but in combination with GCA-F, PA, and Fazekas scores, it is even more prominent for diagnosing DLB patients.

Many studies have confirmed that the core symptoms of DLB can be found in AD. RBD is considered to be associated with α-synuclein related diseases such as DLB, Parkinson's disease (PD) and multiple system atrophy (MSA) (8). However, increasing evidence has shown that RBD is also found in AD (8, 39, 40), but the incidence of RBD in AD patients is significantly lower than that of α-synuclein diseases, even less than 10%, and the occurrence of RBD may increase with the prolonged course of AD patients (39, 40). Visual and auditory hallucinations are also common manifestations in AD, with a prevalence of 10–20% (9, 41). Prior studies suggested that AD patients develop visual hallucinations significantly later than DLB patients, and tend to occur in the advanced stage of AD. In addition, AD patients with visual hallucinations are more likely to develop LB pathology, which also accelerates cognitive decline and increases the mortality risk (9, 42). Cognitive fluctuations can also occur in the normal elderly and AD patients. Escandon et al. (43) found that 12% of AD patients had cognitive fluctuations, and fluctuations exacerbated cognitive performance deterioration. Therefore, in the first interview, visual rating scales may be a better way to help distinguish between the AD and DLB.

Due to the certain subjective evaluation of the visual rating scales, with the development of quantitative detection methods such as manual delineation of volume and automatic analysis, some studies have also been compared. Westman et al. (30) compared the accuracy of the MTA visual rating assessment, automated regional volume and cortical thickness measures and showed that the predictive accuracy of the MTA visual rating assessment was similar to automated regional volume, and both accuracies are comparable to manual hippocampal volume measurements. Both measurements have a sensitivity of over 75% and a specificity of over 80%; Similarly, in the PA and GCA-F visual rating scales, some studies have demonstrated a good correlation between the PA visual rating scale with the automated tool. In the meantime, the different severity scores in the rating scale corresponded to different quantitative degrees of atrophy (44, 45). When comparing frontal lobe atrophy, Ferreira et al. (33) measured used fully automated and manual tracings quantitative imaging methods and provided a comprehensive validation with the GCA-F visual rating scale. A strong correlation was found between the GCA-F visual rating scale and quantitative imaging methods, and the GCA-F reliably reflects cortical atrophy in the frontal lobe. Therefore, we think that the visual rating scales can be used as an indirect measure method to assess the quantitative detection of medial temporal lobe, frontal lobe and posterior cortical atrophy. Meanwhile, we also compared the relationship between the Fazekas scale and automatic analysis. Most studies believed that the relationship between the Fazekas scale and the WMH automatic measurement was consistent, and the Fazekas scale showed significant differences in distinguishing AD patients from others (46, 47). The Fazekas scale can also replace automated analytical measures when analyzing the relationship between clinical parameters and WMHs (48, 49). Therefore, the Fazekas scale can indirectly reflect the white matter lesions.

MTA is a recognized feature of AD but is also present in other dementia, such as frontotemporal degeneration lobe degeneration (FTLD), vascular dementia (VaD) and DLB (34, 50). For AD, the typical feature is atrophy of the hippocampus, but it is also accompanied by atrophy of other regions and accelerated whole-brain atrophy rates compared with normal elderly individuals (51, 52). Previous studies have shown that in the early stage of the AD, patients have experienced the faster atrophy of the temporal lobe, cingulate gyrus, and parietal lobe than normal elderly. With the progress of the disease, atrophy of the frontal lobe also accelerates (53–55). Our results are consistent with those of previous studies. Medial temporal lobe atrophy occurs in DLB patients, but DLB patients have more medial temporal lobe preservation than AD patients. Most previous studies have shown that many patients with DLB have AD pathology (5, 56, 57). The extent of atrophy may also influence the subsequent clinical course of DLB, as individuals with higher levels of hippocampal atrophy have shorter survival than those with lower levels of atrophy (22, 58, 59). For AD pathology in DLB patients, an experiment used the PET tracer ^11^C-Pittsburgh compound B to label Aβ deposition in DLB, except for the temporal lobe, Aβ deposition was found to be associated with a higher atrophy rate of the posterior cingulate gyrus and occipital lobe (60). Investigations of tau-related pathology with 18F-AV-1451 have found that DLB patients display increased uptake in the posterior and inferior temporoparietal, occipital and parietal lobes simultaneously (61, 62). At the same time, other studies considered that frontal atrophy in DLB patients is only related to Lewy pathology but not closely related to AD pathology, and compared with AD, there is no difference between frontal gray matter in DLB patients (57, 63). Based on previous studies, our results found the characteristics of brain atrophy in DLB patients with different severity, compared with AD, patients with mild to moderate DLB show preservation of the medial temporal lobe, while atrophy of the frontal and parietal lobes is roughly the same. As the disease progresses, some DLB patients have AD pathological manifestations, with deposition of Aβ and tau proteins, while DLB patients with AD pathology progress more rapidly, and most patients develop moderate to severe disease, especially in the medial temporal lobe (23, 57, 64). At the same time, AD patients continue to progress; therefore, patients with severe DLB have a degree of medial temporal lobe atrophy consistent with that of patients with AD, and the parietal lobe is preserved in severe cases. In addition, combining multiple imaging indicators to diagnose DLB can improve diagnostic performances and have relatively better sensitivity with similar specificity.

In addition, we studied white matter lesions, microbleeds and ventricle conditions in AD, DLB and cognitively normal older adults. We know that many factors may be associated with white matter lesions, such as smoking, hypertension, T2DM, cardiovascular disease, autonomic impairment like orthostatic hypotension and syncope (26, 65, 66). Therefore, our study also examined the prevalence of these potential factors. The results showed that the proportion of these factors in each group was relatively balanced. We found that regardless of the severity, patients with cognitive impairment had more significant white matter lesions than elderly patients with normal cognition, and the degree of white matter lesions in AD patients was more serious than that in DLB patients with the same severity. White matter hyperintensities (WMHs) are a common feature of elderly people and those with cerebrovascular diseases and are highly correlated with age, vascular factors and neurodegenerative disorders (67, 68). WMH can appear in asymptomatic older adults and also in patients with AD and DLB (26, 68). Similar to previous studies, WMH in patients with cognitive impairment is higher than that in normal cognitive subjects (24, 26). However, the impact between AD and DLB patients is still unclear. A novel finding is that for patients with AD and DLB of the same severity, white matter lesions of AD patients are more serious, after excluding the potential factors. Previous autopsy studies have shown that there is a relationship between cortical hyperphosphorylated tau pathology and Aβ deposition with the severity of WMHs. Thus, the presence of AD pathology may aggravate WMHs (25, 69). Furthermore, most studies have found that there is a close connection between microbleeds and WMHs, and high microbleed counts can lead to cognitive decline (70). Our results show that the frequency and number of microbleeds in patients with cognitive impairment are greater than those in normal elderly individuals, but we did not find a significant difference between AD and DLB, which was similar to a previous study (71). Our study did not find differences in ventricular enlargement between different patients.

Our advantage lies in the discovery of certain brain structures in DLB patients of different severities. We advocate that patients with cognitive impairment should be diagnosed as early as possible in the early stage, which can facilitate subsequent early intervention, treatment and prognosis. Only a few patients show all of the core clinical features in the early stage of DLB, which contributed to high specificity but low sensitivity of current DLB criteria, so the clinical diagnosis of DLB is very difficult. Combined with this study, we suggest in DLB patients' first visit, based on neuropsychological assessment, we can make a comprehensive judgment by combining different regional brain atrophy patterns and white matter hyperintensity in MRI. Although there are important findings revealed by our study, there are also limitations. First, the diagnoses of AD and DLB were made according to the present diagnostic criteria based on clinical manifestations and auxiliary examinations. Although some of the patients included in our study were confirmed by PET, not all patients completed PET examinations or received pathological diagnosis support. Second, the clinical sample size was small as 173 AD patients and 97 DLB patients were included in the study. Third, focuses on the people who participated in the assessment for the first time and lacks relevant follow-ups. Fourth, our study only relied on visual rating scales to assess the degree of atrophy and lacked automatic analysis for verification. Future studies need to further improve the automatic analysis results.

## Conclusion

In conclusion, we found that patients with DLB have widespread cortical atrophy. Compared with AD patients, mild to moderate DLB patients had relatively retained medial temporal lobe atrophy, while both had similar frontal and posterior cortex atrophy. At the same severity, white matter hyperintensity is more serious in AD. For better prognosis, combined multiple imaging visual rating scales may be used for the early diagnosis of DLB.

## Data Availability Statement

The raw data supporting the conclusions of this article will be made available by the authors, without undue reservation.

## Ethics Statement

The studies involving human participants were reviewed and approved by the Committee for Medical Research Ethics at Tianjin Huanhu Hospital and the Tianjin Health Bureau. The patients/participants provided their written informed consent to participate in this study.

## Author Contributions

YJ designed the study. HZ, HL, FW, and SL wrote the report. XD and YY did the statistical analyses. ZS, JG, DL, and LW contributed to the interpretation and discussion of results and reviewed the manuscript. All the authors contributed to the collection of clinical data, revision of the manuscript, read, and approved the submitted version.

## Funding

This work was supported by the National Natural Science Foundation (Grant Number 82171182), Science and Technology Project of Tianjin Municipal Health and Health Committee (Grant Number ZC20121 and KJ20048).

## Conflict of Interest

The authors declare that the research was conducted in the absence of any commercial or financial relationships that could be construed as a potential conflict of interest.

## Publisher's Note

All claims expressed in this article are solely those of the authors and do not necessarily represent those of their affiliated organizations, or those of the publisher, the editors and the reviewers. Any product that may be evaluated in this article, or claim that may be made by its manufacturer, is not guaranteed or endorsed by the publisher.
