# Supplementary figures and images for: Characteristics of Cortical Atrophy and White Matter Lesions Between Dementia With Lewy Bodies and Alzheimer's Disease: A Case-Control Study
Source: Front Neurol. 2022 Jan 11;12:779344. doi: 10.3389/fneur.2021.779344 (PMC8788384; doi:10.3389/fneur.2021.779344)

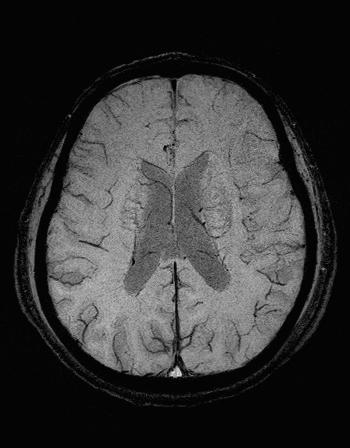

Supplement: Supplementary file 1 [file Data_Sheet_1.ZIP › Supplementary materials(1)/CMBs/CMBs mild.tif]

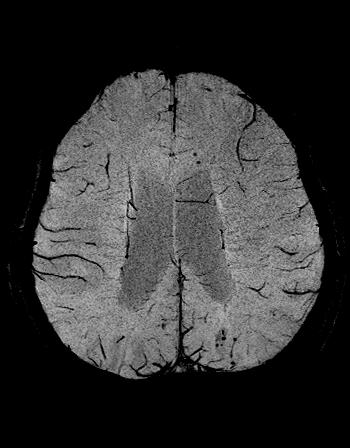

Supplement: Supplementary file 1 [file Data_Sheet_1.ZIP › Supplementary materials(1)/CMBs/CMBs moderate.tif]

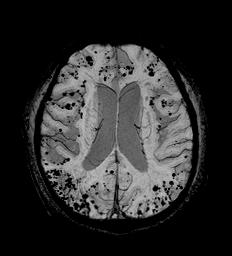

Supplement: Supplementary file 1 [file Data_Sheet_1.ZIP › Supplementary materials(1)/CMBs/CMBs severe.tif]

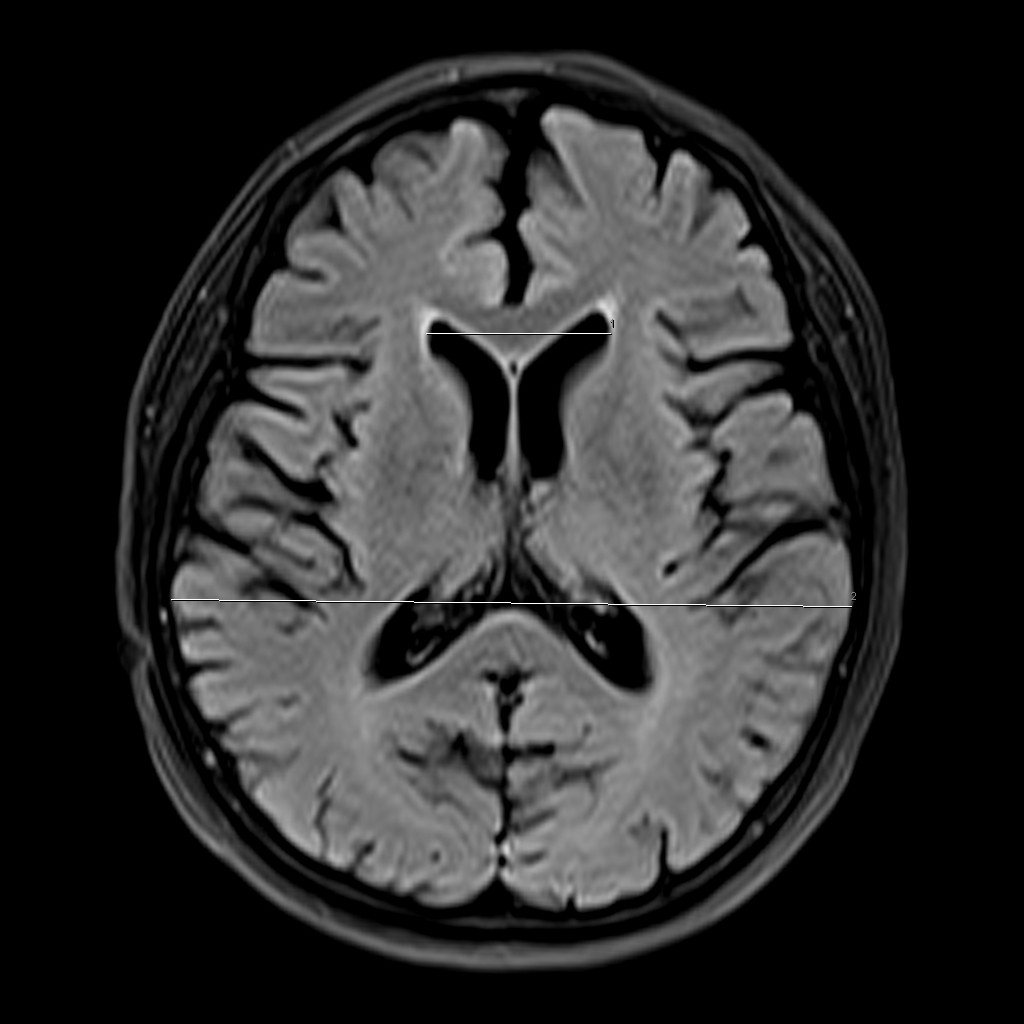

Supplement: Supplementary file 1 [file Data_Sheet_1.ZIP › Supplementary materials(1)/Evans index/Evans Index.tif]

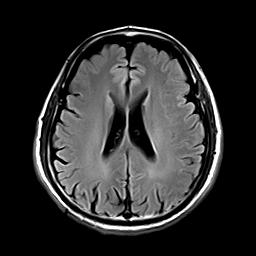

Supplement: Supplementary file 1 [file Data_Sheet_1.ZIP › Supplementary materials(1)/Fazekas/Fazekas 0.tif]

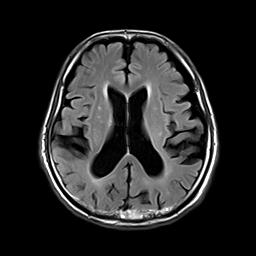

Supplement: Supplementary file 1 [file Data_Sheet_1.ZIP › Supplementary materials(1)/Fazekas/Fazekas 1.tif]

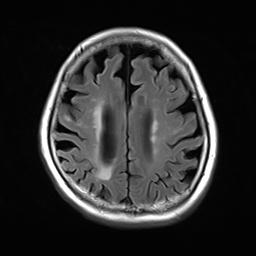

Supplement: Supplementary file 1 [file Data_Sheet_1.ZIP › Supplementary materials(1)/Fazekas/Fazekas 2.tif]

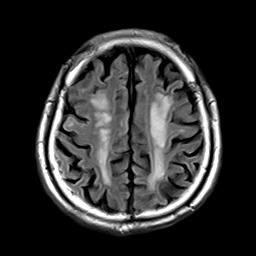

Supplement: Supplementary file 1 [file Data_Sheet_1.ZIP › Supplementary materials(1)/Fazekas/Fazekas 3.tif]

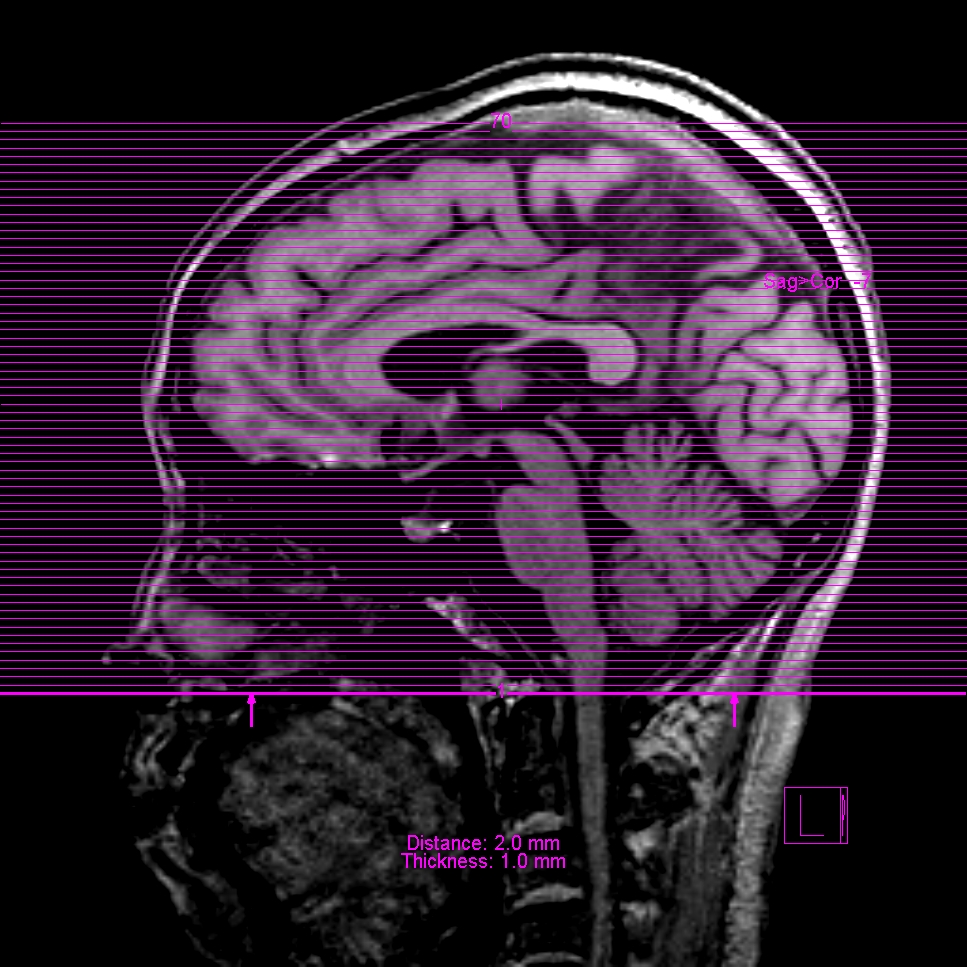

Supplement: Supplementary file 1 [file Data_Sheet_1.ZIP › Supplementary materials(1)/GCA-F/GCA-F positioning phase.tif]

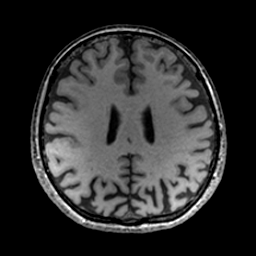

Supplement: Supplementary file 1 [file Data_Sheet_1.ZIP › Supplementary materials(1)/GCA-F/GCA-F2.tif]

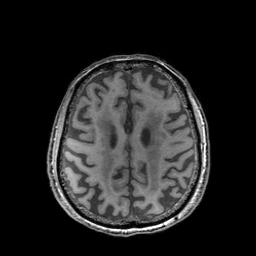

Supplement: Supplementary file 1 [file Data_Sheet_1.ZIP › Supplementary materials(1)/GCA-F/GCA-F3.tif]

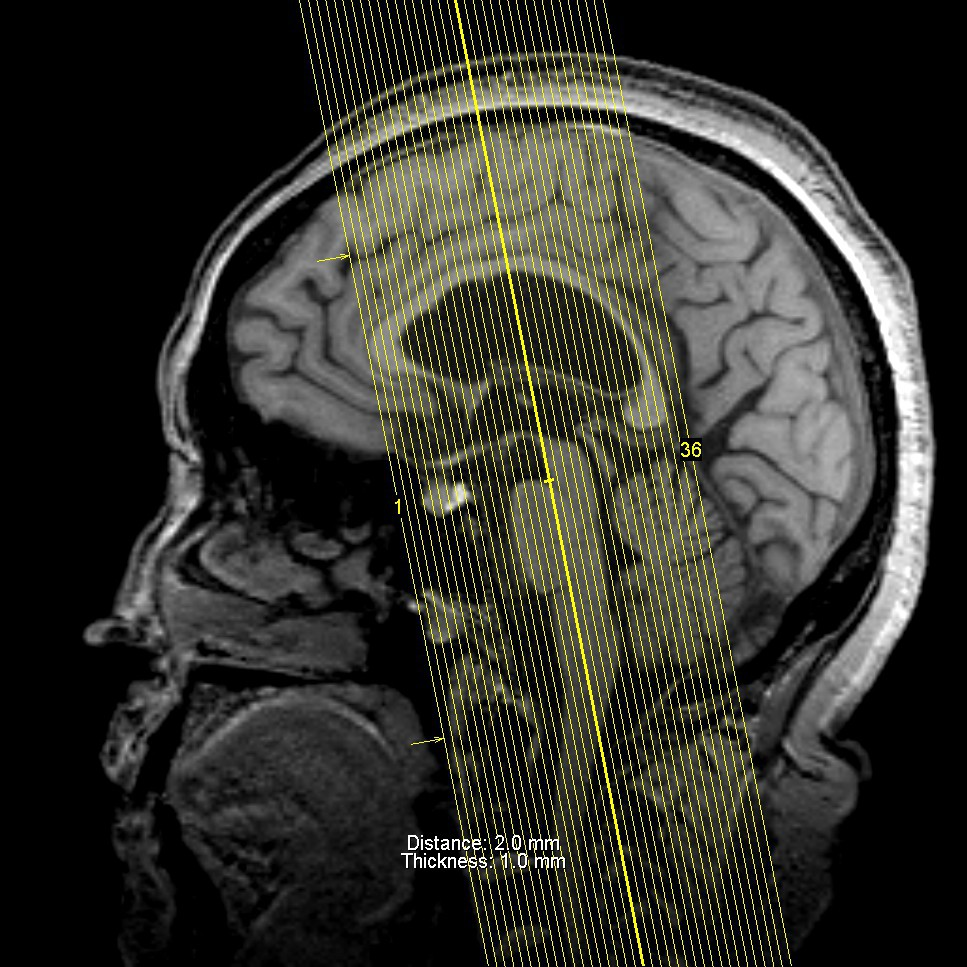

Supplement: Supplementary file 1 [file Data_Sheet_1.ZIP › Supplementary materials(1)/MTA/MTA positioning phase.tif]

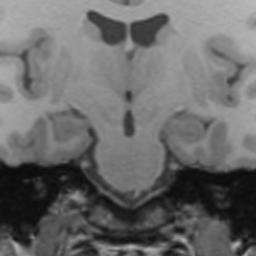

Supplement: Supplementary file 1 [file Data_Sheet_1.ZIP › Supplementary materials(1)/MTA/MTA0.tif]

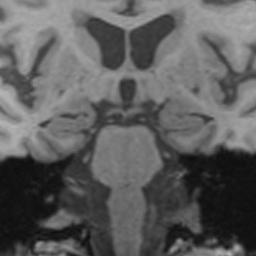

Supplement: Supplementary file 1 [file Data_Sheet_1.ZIP › Supplementary materials(1)/MTA/MTA1.tif]

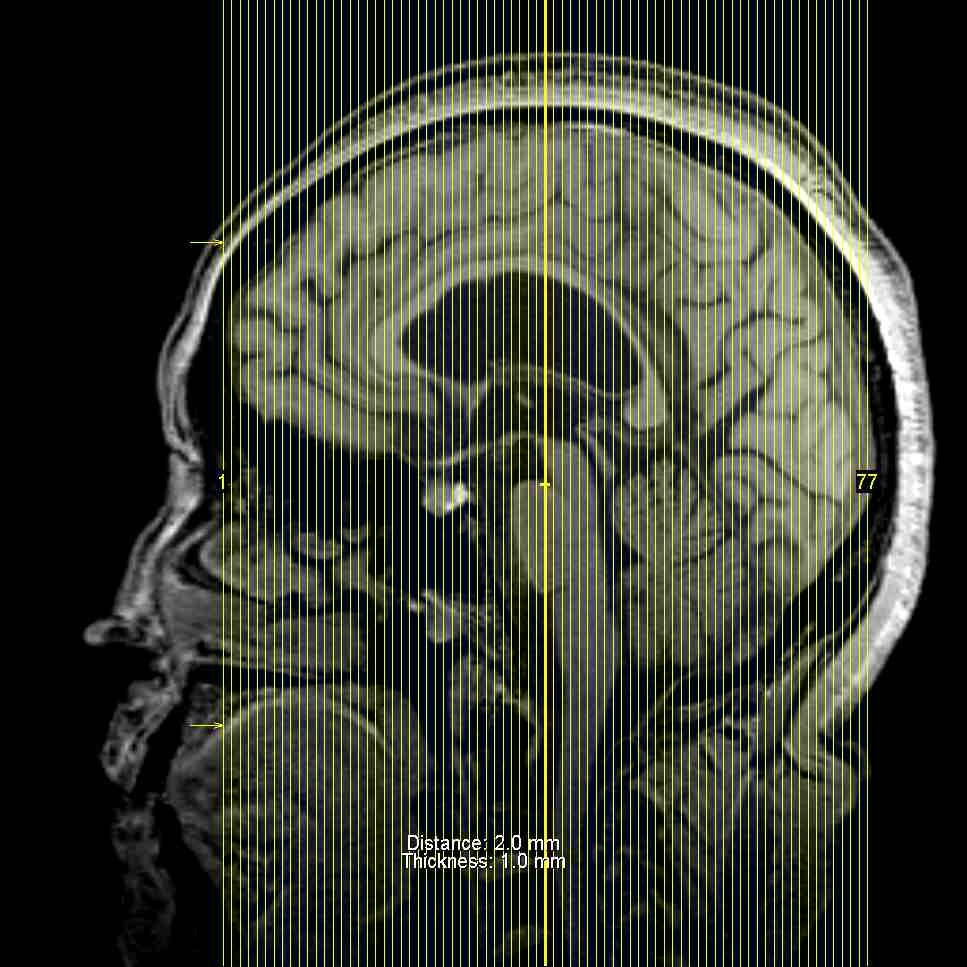

Supplement: Supplementary file 1 [file Data_Sheet_1.ZIP › Supplementary materials(1)/PA-COR/PA-COR positioning phase.tif]

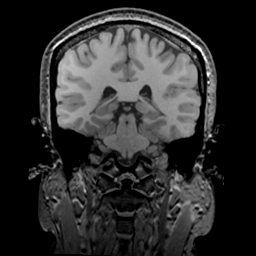

Supplement: Supplementary file 1 [file Data_Sheet_1.ZIP › Supplementary materials(1)/PA-COR/PA-COR0 .tif]

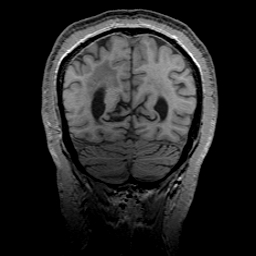

Supplement: Supplementary file 1 [file Data_Sheet_1.ZIP › Supplementary materials(1)/PA-COR/PA-COR1.tif]

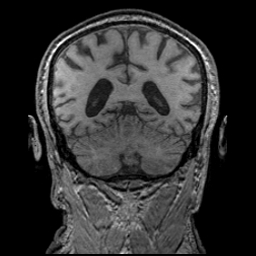

Supplement: Supplementary file 1 [file Data_Sheet_1.ZIP › Supplementary materials(1)/PA-COR/PA-COR2.tif]

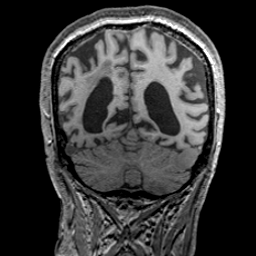

Supplement: Supplementary file 1 [file Data_Sheet_1.ZIP › Supplementary materials(1)/PA-COR/PA-COR3.tif]

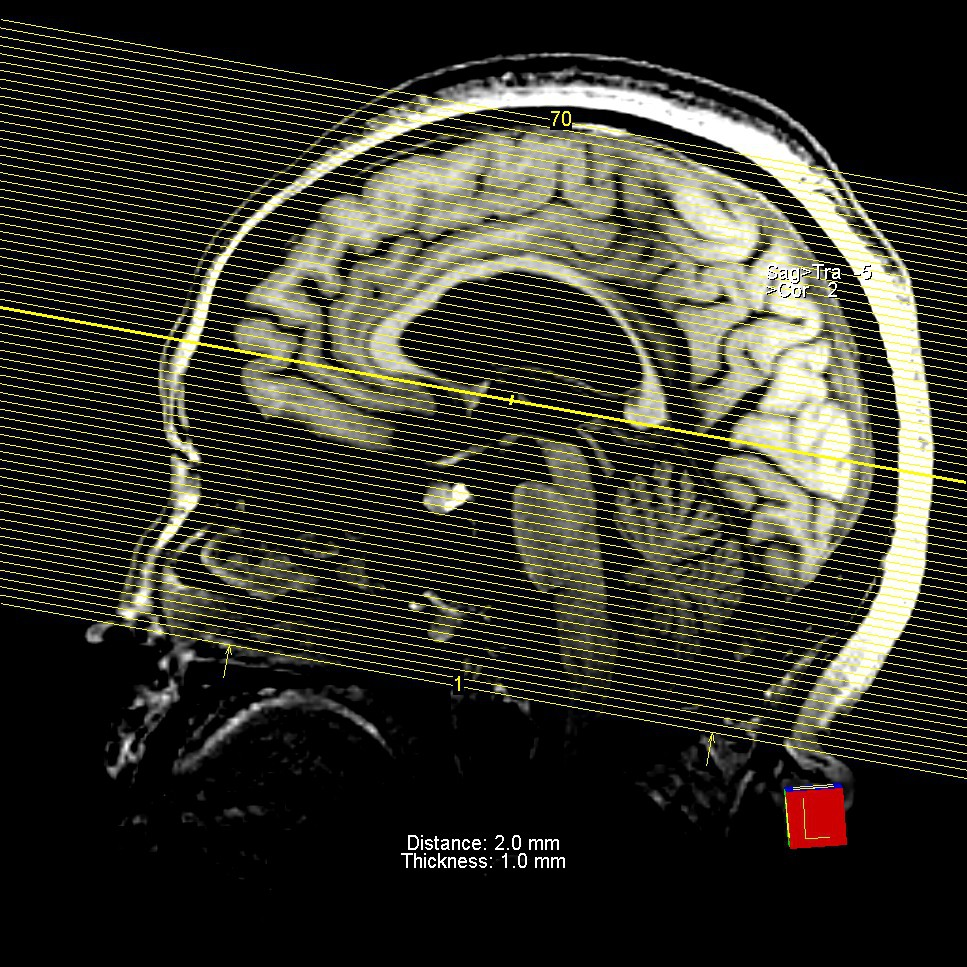

Supplement: Supplementary file 1 [file Data_Sheet_1.ZIP › Supplementary materials(1)/PA-TRA/PA-TRA positioning phase.tif]

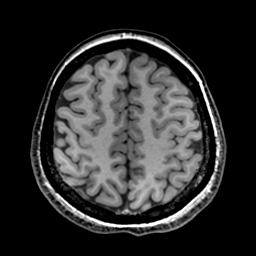

Supplement: Supplementary file 1 [file Data_Sheet_1.ZIP › Supplementary materials(1)/PA-TRA/PA-TRA0.tif]

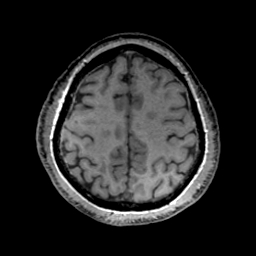

Supplement: Supplementary file 1 [file Data_Sheet_1.ZIP › Supplementary materials(1)/PA-TRA/PA-TRA1.tif]

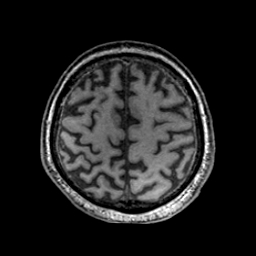

Supplement: Supplementary file 1 [file Data_Sheet_1.ZIP › Supplementary materials(1)/PA-TRA/PA-TRA2.tif]

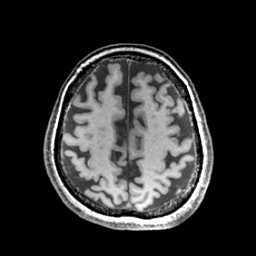

Supplement: Supplementary file 1 [file Data_Sheet_1.ZIP › Supplementary materials(1)/PA-TRA/PA-TRA3.tif]
